# The Dual Role of Mesenchymal Stem Cells in Cancer Pathophysiology: Pro-Tumorigenic Effects versus Therapeutic Potential

**DOI:** 10.3390/ijms241713511

**Published:** 2023-08-31

**Authors:** Youssef Slama, Franck Ah-Pine, Mohamed Khettab, Angelique Arcambal, Mickael Begue, Fabien Dutheil, Philippe Gasque

**Affiliations:** 1 Unité de Recherche Études Pharmaco-Immunologiques (EPI), Université de La Réunion, CHU de La Réunion, Allée des Topazes, 97400 Saint-Denis, La Réunion, France; franck.ahpine@gmail.com (F.A.-P.); mkhettab94@gmail.com (M.K.); philippegasque@gmail.com (P.G.); 2 Service de Radiothérapie, Clinique Sainte-Clotilde, Groupe Clinifutur, 127 Route de Bois de Nèfles, 97400 Saint-Denis, La Réunion, France; mickael.begue@clinifutur.net (M.B.); fabien.dutheil@clinifutur.net (F.D.); 3 Laboratoire Interdisciplinaire de Recherche en Santé (LIRS), RunResearch, Clinique Sainte-Clotilde, 127 Route de Bois de Nèfles, 97400 Saint-Denis, La Réunion, France; angeliquearcambal@gmail.com; 4 Service d’Anatomie et Cytologie Pathologiques, CHU de La Réunion sites SUD—Saint-Pierre, Avenue François Mitterrand, 97448 Saint-Pierre Cedex, La Réunion, France; 5 Service d’Oncologie Médicale, CHU de La Réunion sites SUD—Saint-Pierre, Avenue François Mitterrand, 97448 Saint-Pierre Cedex, La Réunion, France

**Keywords:** mesenchymal stem cells, stroma, tumor microenvironment, therapies, cancer

## Abstract

Mesenchymal stem/stromal cells (MSCs) are multipotent cells involved in numerous physiological events, including organogenesis, the maintenance of tissue homeostasis, regeneration, or tissue repair. MSCs are increasingly recognized as playing a major, dual, and complex role in cancer pathophysiology through their ability to limit or promote tumor progression. Indeed, these cells are known to interact with the tumor microenvironment, modulate the behavior of tumor cells, influence their functions, and promote distant metastasis formation through the secretion of mediators, the regulation of cell–cell interactions, and the modulation of the immune response. This dynamic network can lead to the establishment of immunoprivileged tissue niches or the formation of new tumors through the proliferation/differentiation of MSCs into cancer-associated fibroblasts as well as cancer stem cells. However, MSCs exhibit also therapeutic effects including anti-tumor, anti-proliferative, anti-inflammatory, or anti-oxidative effects. The therapeutic interest in MSCs is currently growing, mainly due to their ability to selectively migrate and penetrate tumor sites, which would make them relevant as vectors for advanced therapies. Therefore, this review aims to provide an overview of the double-edged sword implications of MSCs in tumor processes. The therapeutic potential of MSCs will be reviewed in melanoma and lung cancers.

## 1. Introduction

Mesenchymal stem/stromal cells (MSCs) have aroused great interest over the past 20 years. This interest is partly due to their implication in various physiological and pathological processes, comprising development, tissue repair, organ transplantation, auto-immunity, and cancer [1,2,3,4,5,6,7,8,9,10]. Initially, MSCs were isolated from adult’s bone marrow (BM) in the late 1960s and were defined as fibroblast-like cells [11,12]. Caplan and co-authors qualified them as “mesenchymal stem cells” in 1991 for their capacities of self-renewal and multilineage differentiation [13,14]. Indeed, MSCs can give rise to several mesenchymal tissues by differentiation into several cell types [15,16,17]. It is based on a publication of the International Society for Cellular Therapy (ISCT) from 2006 that MSCs were redefined.

The ISCT issued recommendations, in particular related to the designation of these cells as “multipotent mesenchymal stromal cells” [18,19]. However, the most commonly used terms remain “mesenchymal stromal cells” and “mesenchymal stem cells”. Thus, MSCs are named differently depending on their tissue location. Despite them having been initially described in BM, they were also isolated from many other tissues in different proportions [20,21,22,23,24]. Indeed, MSCs were detected in adipose [25,26], pulmonary [27], synovial [28], renal [22], hepatic [29], bone [30], and muscle [31,32] tissues as well as in the umbilical cord [3,4,5,6,7,8,9,10,11,12,13,14,15,16,17,18,19,20,21,22,23,24,25,26,27,28,29,30,31,32,33,34,35], placenta [36,37], or peripheral blood [33,38,39]. Most studies defined MSCs according to the minimum criteria proposed by the ISCT obtained on BM-derived MSCs, including cell properties to adhere to plastic under standard culture conditions, the expression of surface markers such as CD105 (Endoglin, TGFβR), CD73, and CD90 (Thy-1), the absence of surface markers such as CD45, CD34, CD14, CD11b, CD19, CD79a, and HLA-DR, as well as their plasticity as presented in Table 1 [40,41]. Plasticity is measured through the ability of MSCs to differentiate into osteocytes, adipocytes, and chondrocytes in response to appropriate growth factors under in vitro conditions [19]. 

Of note, there are substantial functional and phenotypic differences between MSCs depending on their tissue sources [42]. However, even today, a complete and precise definition for MSCs’ function and role is still missing. Unlike hematopoietic cell subpopulations, whose identity, developmental stage, and plasticity can be predicted based on the expression of transcription factors and a combination of cell surface markers, MSCs lack specific markers [43,44,45,46]. Consequently, the currently used definitions and classifications for MSCs are based on phenotypic and functional criteria common to other cell types, which are not specific enough. Of note is that MSCs may display several differentiation properties. Indeed, growth factors could promote the differentiation of a fraction of MSCs into osteocytes, adipocytes, or chondrocytes, leading to functional heterogeneity despite the expression of identical surface markers [47,48].

The literature data largely demonstrated that MSCs can both promote and limit tumor growth, with a pro-tumor predominance as recently described by Galland et al. [49]. In this present review, we aim to first review the function and role of MSCs in cancer progression and, secondly, the potential of MSCs as therapeutic tools.

## 2. Origin of Mesenchymal Stem Cells and Other Stromal Cells in the Tumor Microenvironment

The heterogeneity of the tumor microenvironment (TME) characterizes the tumor properties [50]. The TME that directly influences cancer progression is represented by a heterogeneous group of resident and infiltrating cells, which interact together through the secretion of mediators and the production of extracellular matrix (ECM) in order to support the dynamic ecosystem of the tumor [51]. Tumor parenchyma cells grow symbiotically by interacting with the surrounding stroma through paracrine and juxtacrine signaling events.

Cells and molecules composing the TME constitute a complex network involving cellular and physical changes within host tissue to support tumor growth and cancer progression [52,53]. The TME represents the non-neoplastic compartment surrounding tumor tissues. The exact composition of the TME varies depending on the type of tumors considered, but it notably comprises immune cells such as tumor-associated macrophages (TAMs), pericytes (PCs)/vascular smooth muscle cells (vSMCs), endothelial cells, myofibroblasts, sometimes adipocytes, MSCs, and fibroblasts, which are the most abundant cell type in the tumor microenvironment, also called tumor-associated fibroblasts (TAFs) or cancer-/carcinoma-associated fibroblasts (CAFs) [10,54].

**Figure 1:**
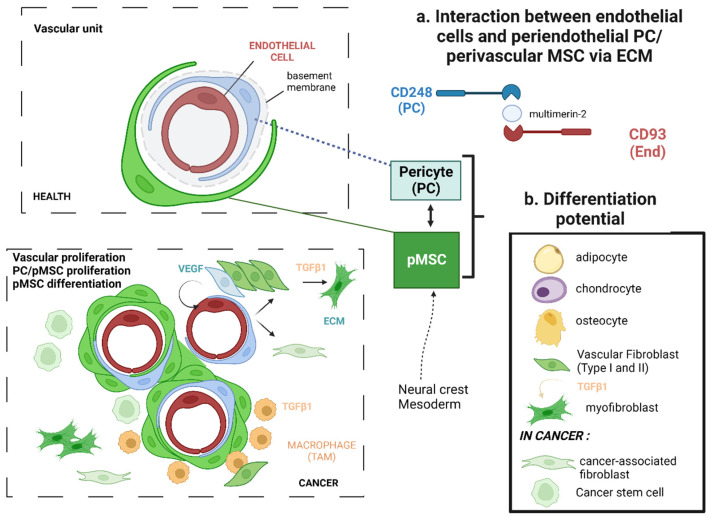
The proliferation and differentiation of perivascular (p) MSCs in cancer can give rise either to pericytes, perivascular fibroblasts, cancer-associated fibroblasts, as well as cancer stem cells. The vascular unit is formed by endothelial cells (CD93+), peridentothelial pericytes (PCs, CD248^high^) embedded into the extracellular matrix (ECM, e.g., multimerin-2) of the basement membrane, and perivascular mesenchymal stem cells (pMSCs CD248^low^/CD90+). Single-cell RNA profiling experiments have indicated that PCs (e.g., in the CNS) may give rise to subsets of pMSCs, which can proliferate, particularly in response to PDGF, and differentiate into either fibroblasts subsets (ECM^high^ or VEGF^high^), cancer-associated fibroblasts (CAFs to support tumor growth), or cancer stem cells (CSCs), as recently evidenced in glioblastoma [63,73]. Fibroblasts stimulated by TGF-β1 may give rise to myofibroblasts (Smooth Muscle cell Actin/SMA^high^/ECM^high^). TAMs: tumor-associated macrophages. (Figure designed by BioRender).

MSCs in adult tissues have two main embryonic origins, deriving either from the mesoderm or the ectoderm neural crest cells, for example, in the central nervous system (CNS) [20,21,55,56,57]. During embryogenesis, MSCs can migrate along vessels and then reside in perivascular (p) niches to give rise to pMSCs in adult tissues, adopting almost similar features and cell surface markers to those of PCs (Figure 1) [21,58,59]. MSCs can also migrate along axons and express peripheral myelin markers, such as myelin P zero or myelin P zero-like 2 (MPZL2), generally found on Schwann cells of the peripheral nerves.

Commonly applied markers or genes (Table 1 and Table 2), such as NG2/Cspg4, CD13/Anpep, and desmin, are not specific to MSCs and their expression is not stable, particularly in disease conditions. Other markers, like CD248 (endosialin, also known as tumor endothelium marker 1, TEM1) and CD90/THY-1, are highly expressed by PCs but also by pMSCs, especially in the context of cancer [60,61]. It is established that PCs expressing high levels of CD248 maintain the interaction with CD93 on endothelial cells via the ECM protein multimerin-2 (Figure 1) [62].

Recent studies utilizing cell lineage tracing and single-cell RNA sequencing experiments have provided insights into the different cell types present in the stroma of healthy tissues (lung, heart, brain, skin, and colon) and the TME [60,63,64,65]. These studies have recently been reviewed comprehensively by Betsholtz et al. in 2022 [66]. All these studies support the emerging notion that periendothelial cells such as PCs (in close association with the basement membrane of endothelial cells) can give rise to pMSCs detaching from the vessels and infiltrating the tissue parenchyma [63,67]. Inversely, in zebrafish, pMSCs have been shown to give rise to PCs to support vessel survival/growth [68]. Interestingly, Kalluri and colleagues have long argued that the distinction between fibroblasts and MSCs is extremely difficult and the immunological and molecular collective evidence suggest that quiescent or resting fibroblasts are precursors of activated fibroblast called MSCs [69]. In the brain, this has been further evidenced by Garcia and colleagues, observing that PCs and MSCs (called vascular fibroblasts by the authors) have similar transcriptomic signatures [63].

Resting MSCs have the potential to proliferate when stimulated by growth factors, for example, transforming growth factor-β (TGF-β), platelet-derived growth factor (PDGF), and interleukin-6 (IL-6). Properties assigned to stromal fibroblasts are in fact properties associated with ‘activated’ fibroblasts, myofibroblasts, and MSCs. Once activated, their functions include the production of ECM cytokines and chemokines, recruiting immune cells, and exerting physical forces to modify tissue architecture. PCs maintain vascularization by providing structural support to blood vessels [59]. Their abundance in tumor stroma is directly related to their physiological dysregulation. Indeed, they constitute the pre-metastatic niche and participate in immunosuppression through M2 macrophage polarization and the growth of cancer cells [59,60,61,62,63,64,65,66,67,68,69,70]. Of note is that the PCs’ detach from endothelial cells to give rise to proliferating and infiltrating MSCs, which may help to promote tumor cell invasion (Figure 1) [71]. Angiogenesis is regulated by PCs/MSCs that mediate pro-angiogenic mediator secretions (e.g., VEGF).

Hence, during cancer development, cells composing the TME interact directly or indirectly with cancer cells and ECM through the secretion of pro-inflammatory cytokines, chemokines, and growth factors, in order to support tumor growth, proliferation, and metastatic tumor formation [72]. These interactions associated with pro-inflammatory secretions promote the alteration of the original properties and functions of TME partners. Then, new partners such as immune and stromal cells are recruited and come to enrich this complex network.

**Table 2:** Canonical cell markers in healthy cells and in the tumor microenvironment.

Cell Types	Markers	Comments	References
Endothelial cells	**CD31**	PECAM-1, homotypic cell adhesion	[60]
	**CD93**	CD248 family member	[60,74]
	CLDN5	Claudin 5	[60]
	CDH5	Cadherin 5	[60]
Pericytes (PCs)	**PDGFRβ**	Receptor for platelet-derived growth factor	[59]
	**NG2**	Encoded by Chondroitin sulfate proteoglycan 4 *CSPG4* gene.	[59,60]
	Desmin		[59]
	CD13	Alanyl Aminopeptidase N	[59]
	**CD248**	Endosialin (TEM-1), highly expressed in tumor tissues (PCs > pMSCs)	[59,61,75]
	**KCNJ8**	Kir6.1, Potassium inwardly rectifying channel, associates with Abcc9	[59,60,76]
Vascular smooth muscle cells (vSMCs)	PDGFRβ	Levels in PCs > vSMCs	[77]
	NG2		[77]
	Desmin	Muscle class III intermediate filament	[77]
	CD13		
	RGS5	Regulator of G protein signaling 5 GTPase activating protein	[59,64,78]
	CD146	Melanoma cell adhesion molecule (MCAM)	[59,78]
	**α-** **SMA**	Alpha smooth muscle cell actin encoded by *ACTA2*. Level of expression in vSMCs >> PCs	[59]
	**TAGLN**	Transgelin, smooth muscle protein 22 alpha (SM22).	[59]
Perivascular MSCs (also known as vascular fibroblasts)	VIM FSP FAP PDGFRβ	Vimentin, intermediate-filament ass. protein intermediate-filament ass. protein Fib activation protein alpha, serine protease	[79]
	**PDGFRα**		[60]
	CD13		[79]
	**COL1A1**	pMSCs also express high levels of COL1A2, COL3A1, and COL4A1. Much less expressed by PCs’	
	**LAMA-1**	laminin subunit alpha 1, also expressed by epithelial cells	[60]
	LUM	Lumican	[60]
	DCN	Decorin	[60]
	MPZL2	Myelin protein zero-like 2, adhesion	[60]
	SRPX2	Sushi repeat-containing protein X-linked 2	[60]
	FN	Fibronectin, also expressed by PCs/scar tissue	[80]
Tumor-associated Macrophages (TAMs)	CD11b/CD18	Complement receptor type 3 involved in phagocytosis of host cell debris	[81,82]
	**CD163**	Scavenger receptor, M2 anti-inflammatory	[83]
	**C1q/C3** **CD68**	Complement factors	[84]

Adapted from Ah-Pine et al., 2023 [67]. Several markers are shared between the different subsets of periendothelial/perivascular cells, while others (in bold) are restricted (but not exclusive) to specific subsets. CD248, also known as TEM1: tumor endothelial marker 1, but subsequently found on pericytes around vessels but not expressed by endothelial cells.

### 2.1. Recruitment of Mesenchymal Stem Cells

As previously described, pMSCs exhibit multilineage differentiation and mainly reside in the perivascular niches of all organs [22,85]. Current evidence suggests that the MSCs present in tumor stroma come from both local and distant tissues [86,87,88]. Considering that MSCs are present in most body tissues, the population of tissue-resident MSCs found in the TME likely represents the first population to occupy the TME and support tumor development [89,90,91]. Subsequent recruitment of the distant MSC population is accompanied by a pro-inflammatory state at the tumor site [92]. This phenomenon, called “homing”, represents the migration of cells to the tissue, to graft themselves there and to exert local functional effects. Furthermore, it is well established that MSCs are recruited to tumor sites to support regenerative activity [93,94,95].

Dvorak defined cancer as “a wound that does not heal”, and MSCs are also recruited at tumor sites for tissue repair [93]. Then, MSCs are regulated by the pro-inflammatory environment of the TME, by mediators secreted by tumor cells, and may adopt a pro-tumorigenic phenotype [91,96]. Of note is that the migration of MSCs to tumor sites was observed in particular in Kaposi’s sarcoma [97]; colorectal tumors [98]; pancreatic tumors [99]; glioma [100]; breast, lung, and melanoma metastatic tumors [101,102]; gastric tumors [103]; melanoma [104]; or even ovarian tumors [105]. Many studies described progressive changes in the tumor stroma with an initial apparent expansion of MSCs which precedes the conversion to malignancy. MSCs are often observed circumscribing early or premalignant lesions.

**Figure 2:**
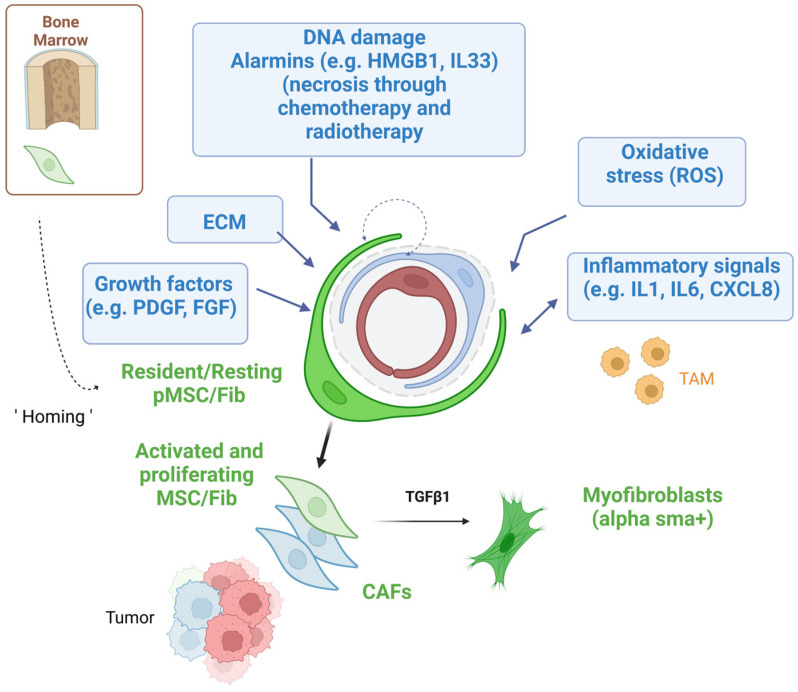
Recruitment of distant (BM)/resident MSCs, activation via diverse mechanisms, and differentiation to CAFs of the TME. Perivascular cells (MSCs/fibroblasts) as well as pericytes (blue) are resident cells, while MSCs from the bone marrow can be recruited in tumor sites. Factors derived mainly from necrotic tissues (notably in response to therapies) and infiltrating immune cells such as macrophages may contribute to the recruitment, proliferation, and activation of MSCs/Fib. MSCs will differentiate into CAFs with either restraining or promoting activities towards cancer cells. (Figure designed by BioRender).

Importantly, during cancer development, MSCs have the ability to migrate and be activated at the tumor sites [86,87,88]. Numerous factors are involved in these migration/activation processes, including growth factors (GF) (Epidermal GF (EGF), stem cell factor (SCF), PDGF, hepatocyte GF (HGF), and insulin GF (IGF-1)) [106,107], angiogenic factors (VEGF, hypoxia-inducible factor (HIF-1α), and Fibroblast GF (FGF-β)) [108,109], inflammatory factors such as chemokines (CXCL8, CCL2, CCL5, CCL22, and SDF-1/CXCL12) [107,108,109,110], and cytokines (TNF-α, TGF-β, IL-6, and IL-1β) [111,112,113,114]. Other factors such as reactive oxygen species (ROS), adhesion molecules (VCAM), matrix metalloproteases (MMPs) of the ECM, alarmins (e.g., HMGB1, ATP, extracellular DNA) produced by necrotic cancer cells, and the level of hypoxia (HIF-1) can also control this migration process (Figure 2) [115,116,117,118,119,120]. Indeed, it is now well established that MSCs express an armory of innate immune receptors (also called pattern recognition receptors, PRRs) for alarmins, which are also able to recognize microbial ligands [24].

### 2.2. Mesenchymal Stem Cell Activation and Multi-Lineage Differentiation

During cancerous pathophysiological process, MSCs are recruited into the TME where they are exposed to multiple pro-inflammatory mediators and other stimuli, which may change their behavior to promote tumor growth through immunomodulation [8].

The MSCs differentiate depending on the tumor stage of development, their tissue origin, the heterogeneity of TME, and the interactions with other cells composing the TME [121].

**Figure 3:**
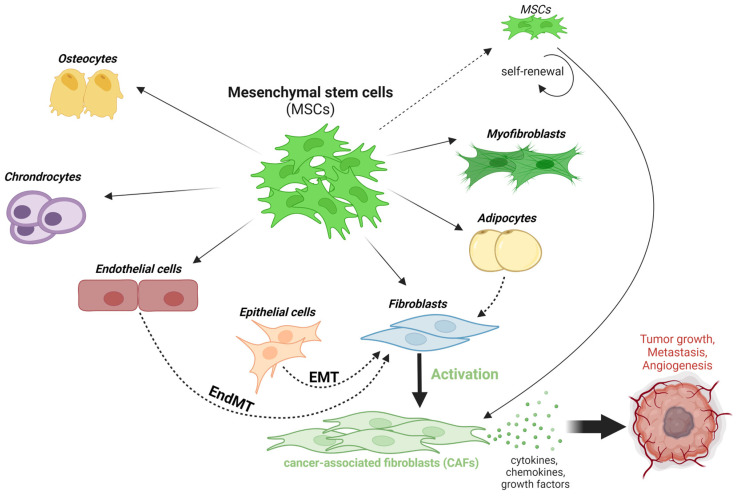
Mesenchymal stem cells are highly plastic and exhibit multipotency (transdifferentiation) in the tumor microenvironment. CAFs: cancer-associated fibroblasts; EMT: epithelial–mesenchymal transition; EndMT: endothelial–mesenchymal transition; MSCs: mesenchymal stem cells. (Figure designed by BioRender).

The MSCs’ lineage differentiation in the TME is presented in Figure 1, Figure 2 and Figure 3. The MSC population can give rise to several cell types, such as fibroblasts. Fibroblasts can differentiate or transdifferentiate into CAFs and play a critical role in cancer progression. The CAFs represent a heterogeneous population of activated fibroblasts, which can also potentially transdifferentiate from epithelial or endothelial cells [122,123].

MSCs are also able to differentiate into adipocytes that may give rise to cancer-associated adipocytes (CAAs) known to play an active role in the TME formation and function [124,125,126]. The CAAs modulate, for instance, the behavior of breast cancer cells via the release of pro-inflammatory mediators such as IL-6, TNF-α, CCL2, or CCL5, promoting angiogenesis, proliferation, and metastasis [126,127,128].

Importantly, MSCs could also give rise to CSCs characterized by their ability to renew, differentiate, mimic the behavior of tumor cells [7,129], or fuse with tumor cells, giving rise to a highly pro-tumor hybrid complex [15,130,131,132]. In the CNS, it has recently been established that PCs/MSCs originating from the neural crest can give rise to CSCs (also known as glioma-stem cells (GSCs)) in high-grade glioma (*Glioblastoma multiforme*) [73].

### 2.3. Interplays between Mesenchymal Stem Cells and Components of the Tumor Microenvironment

To establish the TME and build the primary and secondary sites of the tumor, cells of the TME communicate directly by cell–cell contact or indirectly. The indirect communication between cellular components of the TME contributes to the malignant properties of tumor cells as well as the ECM composition.

**Figure 4:**
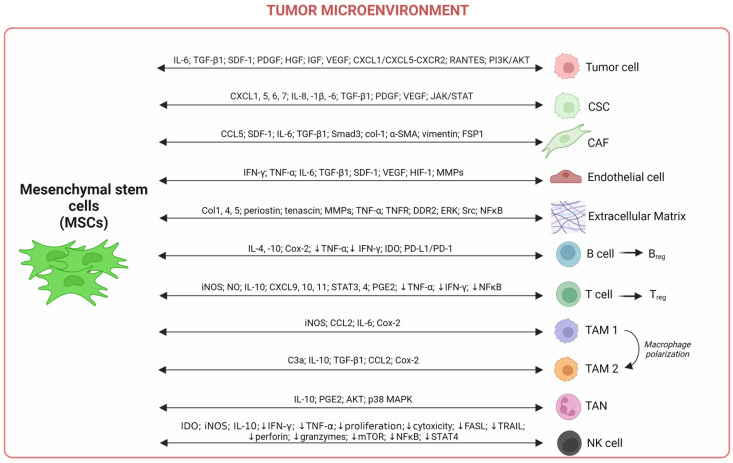
Interactions and regulations involving mesenchymal stem cells in the tumor microenvironment. AKT: protein kinase B; α-SMA: α-smooth muscle actin; C3a: complement component 3 anaphylatoxins; CAF: cancer-associated fibroblast; CCL: CC chemokine ligand; COL: collagen; Cox-2: cyclooxygenase-2; CSC: cancer stem cell; CXCL: C-X-C motif ligand; CXCR: C-X-C motif chemokine receptor; DDR2: discoidin domain receptor tyrosine kinase 2; ERK: extracellular signal-regulated kinase; FASL: fas ligand; FSP1: fibroblast-specific protein 1; HGF: hepatocyte growth factor; HIF-1: hypoxia-inducible factor-1; IDO: indoleamine-2,3-dioxygenase; IFN-γ: interferon-γ; IGF: insulin growth factor; IL: interleukin; iNOS: inducible nitric oxide synthase; JAK/STAT: janus kinase-signal transducer and activator of transcription; MMPs: matrix metalloproteinases; MSCs: mesenchymal stem cells; mTOR: mammalian target of rapamycin; NFκB: nuclear factor-κB; NK: natural killer; NO: nitric oxide; PD-1: programmed cell death protein 1; PDGF: platelet-derived growth factor; PD-L1: programmed cell death ligand 1; PGE2: prostaglandin E2; PI3K: phosphoinositide 3-kinase; RANTES: regulated on activation, normal T cell expressed and secreted; SDF-1: stromal cell-derived factor 1; Smad: small mothers against decapentaplegic; Src: proto-oncogene tyrosine-protein kinase; STAT: signal transducer and activator of transcription; TAM: tumor-associated macrophage; TAN: tumor-associated neutrophil; TGF-β: transforming growth factor-β; TNF-α: tumor necrosis factor-α; TNFR: tumor necrosis factor receptor; TRAIL: tumor-necrosis-factor-related apoptosis-inducing ligand; VEGF: vascular endothelial growth factor. (Figure designed by BioRender).

The pro- or anti-tumorigenic behavior of MSCs is regulated by this environment. As shown in Figure 4, MSCs communicate and regulate the TME components, resident and infiltrating cells promoting cancer progression.

#### 2.3.1. Innate and Adaptative Immune Cells

Among the immune cells present in the TME, selective populations of T and B lymphocytes promote and/or suppress tumor growth [133,134]. A dual role in tumor progression has been attributed to tumor-infiltrating B cells. In the pathophysiology of breast cancer, the literature data reported different conclusions depending on cancer type and stage. In triple-negative breast cancer, a high level of tumor-infiltrating B cells and plasma cells is correlated with a favorable prognosis [135]. The immunosuppressive role of MSCs has already been reported, as well as the effect of T and B cells, as shown in Figure 4. During inflammatory disease, MSCs are known to drive a profile change for B and T cells by inducing regulatory B and T cell production [136]. During prostate cancer, MSCs suppress T cell proliferation, exhibiting immunosuppressive properties, participating in blocking immunologic recognition and the elimination of malignant cells [137]. Interestingly, the B and T cell profiles are influenced by an over secretion of anti-inflammatory mediators, such as IL-4 and IL-10, and a decrease in the secretion of pro-inflammatory mediators (TNF-α, IFN-γ) promoting B and Treg phenotypes [138,139,140]. Moreover, the immunosuppressive role of MSCs on B cells occurs through the modulatory activity of indoleamine-2,3-dioxygenase (IDO), cyclooxygenase-2 (Cox-2), and programmed cell death ligand 1 (PD-L1) [141]. The regulation of T cell activity by MSCs is regulated by iNOS, NO secretion, and prostaglandin E2 (PGE2) [142,143].

TAMs and tumor-associated neutrophils (TANs) are known to secrete pro-inflammatory mediators. Importantly, considering the immune role of macrophages, their capacity to phagocyte, and their cytotoxic properties (through the production of reactive oxygen species (ROS) and complement proteins), they could be associated with anti-tumor immunity. Nevertheless, high levels of TAMs are mainly associated with a poor prognosis in numerous cancers [144]. The recruitment of TAMs into tumors is known to be mediated by the cytokines and chemokines released by tumor cells and CAFs. These factors have been extensively studied and include CCL2, CCL7, glial-cell-line-derived neurotrophic factor (GDNF), VEGF, alarmin ATP (released from necrotic cells), macrophage colony-stimulating factor 1 (CSF1), granulocyte–macrophage colony-stimulating factor (GM–CSF), periostin, and complement anaphylatoxins (e.g., C3a) [145,146,147]. The MSCs promote macrophage polarization in favor of an anti-inflammatory phenotype (TAMs 2). Jia and co-authors found that MSCs express iNOS, CCL2, IL-6, and Cox-2 and recruit macrophages at tumor sites [148]. The IL-6 secreted by MSCs promotes the polarization of infiltrated TAMs into M2-like macrophages that will express an anti-inflammatory profile and play an immunosuppressive role on T cells. Moreover, TAMs participate in the development of metastases [149], while TANs promote angiogenesis and immune suppression [150,151]. MSCs drive TANs to adopt an anti-inflammatory profile and secrete IL-10 and PGE2. MSCs are able to modulate neutrophil activity. In a breast tumor model, an MSC/neutrophil (CD11b+Ly6G+) co-culture model allowed neutrophils to acquire immunosuppressive activity, downregulating T cell proliferation in an in vitro model and amplifying progression tumor in an in vivo model [152]. Then, TANs mediated MSCs’ differentiation into CAFs via the activation of AKT and p38 MAPK signaling pathways [153].

Importantly, the immunosuppressive role of MSCs also affects NK cell activity by decreasing their cytotoxicity capacity through mTOR inhibition in gastric cancer [154]. Consequently, the secretion of granzymes and perforin by NK is downregulated, and that promotes tumor cell survival and cancer progression. Moreover, a study based on a co-culture model of MSCs and NK cells co-exposed to IL-15 demonstrated an alteration of IFN-γ secretion by NK cells, a decrease in their proliferation, as well as a loss of their cytotoxicity linked to the alteration of cytolytic degranulation and perforin release [155]. Interestingly, authors detected important levels of IDO secreted by the MSCs participating in immune response suppression. The decrease in the cytotoxic activity of T and NK cells is also due to an increase in IL-10 and iNOS releases, a decrease in inflammatory effector production (TNF-α, IL-17, FASL, TRAIL), and a lack of immune cell responses to pro-inflammatory cytokines (IL-12, IL-18) leading to a loss of IFN-γ release through NFκB and STAT4 inhibition [138,156,157].

#### 2.3.2. Tumor Cells

Interestingly, on tumor sites, MSCs interact with tumor cells to support tumor development. MSCs communicate with tumor cells indirectly through the secretion of cytokines, chemokines, and growth factors [52,158,159,160]. Among the indirect interactions, there are also interactions via metabolites, such as PGE2 in lymphoblastic leukaemia cells [161]. Importantly, numerous studies are interested in interactions via microparticles, such as functional mRNAs, proteins, or micro-RNAs called “miRs” or “miRNAs”. The MSCs released, via exosomes, miRNAs, play a crucial role in the regulation of cell death. Indeed, it has been demonstrated that the immunoregulatory functions of MSCs could be attributed to miRs such as miR-21 [162,163,164]. Direct interactions occurring between MSCs and tumor cells comprise interactions via Notch signaling, the formation of nanotubes, the fusion of cells, trogocytosis, or Gap junctions permitting exchanges of second messengers such as c-AMP, miRs, or ions [130,165]. Rafii et al. reported trogocytosis, which is an exchange of plasma membrane fragments between ovarian cancer cells and stromal cells, for the first time and demonstrated that it promoted the acquisition of chemoresistance [166].

The crosstalk between MSCs and tumor cells also involves the activation of pro-inflammatory and survival-signaling pathways, such as the NFκB, PI3K/Akt, Wnt, MAPK, and JAK/STAT signaling pathways that mediate tumor progression, metastasis formation, and angiogenesis [120,167,168]. Accordingly, the activation of the NFκB and PI3K/AKT signaling pathways promote immune suppression, EMT, and metastasis in breast and gastric cancers [169,170,171]. In the TME, naive MSCs exhibiting anti-tumor properties are reprogrammed by cancer cells and the TME into CAFs, which participate in tumor progression [172]. Interestingly, CAFs promote tumor growth, angiogenesis, immunosuppression, and metastasis via the secretion of growth factors such as VEGF [173,174], TGF-β [175], cytokines and chemokines (IL-6, CXCL12) [123,176,177], PDGF, and MMPs [178,179,180]. The CSCs are also regulated by MSCs through the secretion of pro-inflammatory mediators as well as growth factors [138,139,140,141,142,143,144,145,146,147,148,149,150,151,152,153,154,155,156,157,158,159,160,161,162,163,164,165,166,167,168,169,170,171,172,173,174,175,176,177,178,179,180,181].

#### 2.3.3. Extracellular Matrix

The TME also comprises non-cellular components, such as ECM as collagen produced abundantly by MSCs [60]. This ECM is crucial for interactions between cellular components of the TME and for cancer expansion [181,182]. Indeed, ECM provides a structural backbone for the tumor stroma as well as active soluble factors such as growth, angiogenic factors, cytokines, chemokines, and hormones secreted by stromal and tumor cells regulating tumor behavior. An in vitro study on a three-dimensional model reported that ECM heterogeneity highly influenced invasion and metastasis efficiency [183]. During the process of tumor development, the ECM is disorganized and collagen deposit as well as cross-linking with other matrix proteins such as elastin, laminin, or fibronectin are associated with metastatic tumor formation [184]. However, depending on the ECM organization and composition, tumor cells will adopt different behavior and cancer will develop in different ways. All the interactions between cells and TME will be directly affected by ECM organization and composition [185,186]. More importantly, recruited immune cells’ behavior will be directly modulated by the ECM composition. Gonzalez et al. demonstrated in human MSCs isolated from breast cancer metastasis that stromal cells expressing a fibroblastic phenotype were able to upregulate collagen receptor expression, namely discoidin domain receptor 2 (DDR2), in breast cancer cells and increase collagen deposits promoting breast cancer cell alignment, migration, and metastasis [187]. Moreover, MSCs also modulate collagen 1, 4, 5, tenascin, and periostin deposits and modulate the ECM composition involving the ERK, Src, and NFκB signaling pathways [138,143,188]. High levels of MMPs expressed by MSCs/fibroblasts in pathological conditions will contribute to ECM degradation and facilitate the invasive behavior of tumor cells [168]. Importantly, as described by Gattazzo and co-authors, the ECM modulates stem cell differentiation and proliferation [185]. The molecular composition of the ECM constitutes an appropriate niche for stem cells that is directly regulated by mediators composing the ECM. The ECM is also regulated by cancer cells and can in turn regulate the behavior of MSCs.

## 3. Role of MSCs-1 and MSCs-2 Subtypes in Cancer Progression

The literature is extensive on the ability of MSCs to develop pro- and anti-tumor phenotypes [189,190]. Their role in tumor progression depends on the balance between their pro- and anti-inflammatory phenotypes [191]. Waterman and co-authors suggested that MSCs could undergo a functional switch from an anti-tumorigenic state, called MSCs-1, to a pro-tumorigenic state, called MSCs-2 [192,193].

Importantly, the immunosuppression capacity of MSCs involves the MSCs-2 phenotype that inhibits immune cells and turns off their anti-tumorigenic properties. Of note is that MSCs-1 inhibits tumor growth by promoting the infiltration of monocytes, granulocytes, and T cells into tumors, contributing to anti-cancer immunity. These immune cells, coupled with the pro-inflammatory state generated in the tumor parenchyma, induce the secretion of chemokines attracting activated lymphocytes to promote further anti-cancer immunity [194]. It has been demonstrated in an in vivo study on Kaposi’s sarcoma that MSCs were able to suppress tumor growth by inhibiting the Akt signaling pathway of tumor cells [97]. The MSCs were also able to suppress tumor proliferation in breast cancer through the inhibition of the Wnt pathway involved in tumor development [195]. Accordingly, studies demonstrated that MSCs upregulated the mRNA expression of a negative regulator of the cell cycle, namely p21, and caspase-3, which is a key protease of apoptosis activation, in tumor cells. This regulation is implicated in the inhibition of tumor cell growth through apoptosis activation and cell cycle arrest in the G(0)/G(1) phase of tumor cells [196]. Additionally, MSCs caused the inhibition of tumor angiogenesis through endothelial cell apoptosis and capillary degeneration, altering tumor growth [77]. Thus, MSCs produced exosomes which blocked hepatocellular carcinoma stem cell malignancy via the upregulation of long non-coding RNAs (IncRNAs), namely C5orf66-AS. Authors demonstrated that C5orf66-AS1 upregulated DUSP1 expression through the inhibition of the microRNA-127-3p (miR-127-3p) production controlled by ERK, significantly reducing the proliferation, migration, invasion, angiogenesis-stimulating, and self-renewal abilities of CSCs [197].

**Figure 5:**
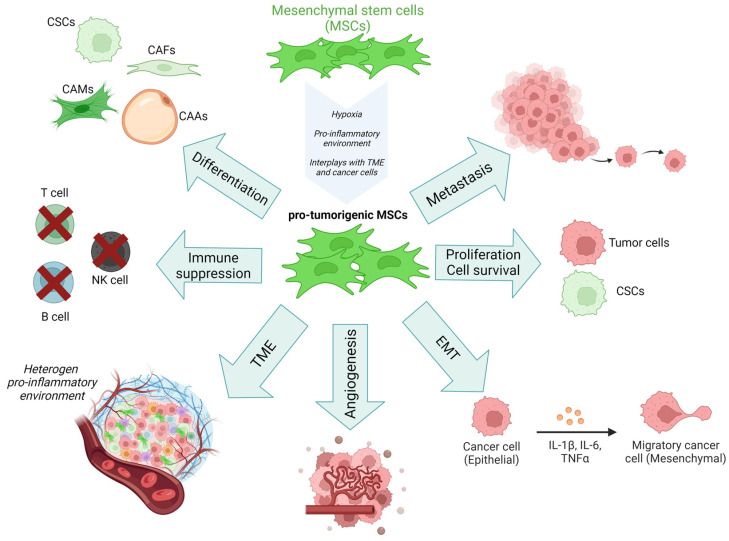
Tumor support functions of mesenchymal stem cells. Adapted from Frisbie et al. [143]. CAAs: cancer-associated adipocytes; CAFs: cancer-associated fibroblasts; CAMs: cancer-associated myofibroblasts; CSCs: cancer stem cells; EMT: epithelial–mesenchymal transition; IL: interleukin; MSCs: mesenchymal stem cells; NK cells: natural killer cells; TME: tumor microenvironment; TNF-α: tumor necrosis factor-α. (Figure designed by BioRender).

The deleterious role of MSCs in the tumorigenesis process is often attributed to diverse mechanisms promoted by MSCs-2 [198]: differentiation into pro-tumorigenic mediators; the suppression of the immune response [199]; angiogenesis [200,201]; EMT; the formation of CSCs; the tumor cells’ survival; and the metastatic process [6,172,202]. All these mechanisms, summarized in Figure 5, are driven by direct and indirect interplays between MSCs, cancer cells, and the TME and promote the resistance of tumor cells to therapeutic treatment [203].

### 3.1. The Epithelial-Mesenchymal Transition

Among the growth factors secreted by TME components, we find TGF-β. This growth factor is a cytokine regulating the cell proliferation, migration, and differentiation of a large number of cell types. At the onset of tumorigenicity, this growth factor acts as a tumor suppressor by inducing cytostasis and apoptosis of normal and pre-malignant cells. However, at an advanced stage, cancer cells become resistant to its cytostatic effects. By directly interacting with the cancer cells, TGF-β can promote tumorigenicity by inducing EMT [204]. In physiological conditions, EMT plays a crucial role in embryogenesis and tissue damage repair [205]. Nevertheless, this process can stimulate tumor development [206]. The EMT is characterized by changes in the production of three prominent biomarkers (E-cadherin, vimentin, and N-cadherin), and these changes can lead to decreased cell adhesion, the loss of tight junctions, and polarity. At the same time, epithelial cells adopt characteristics of a mesenchymal phenotype, including motility and the potential for invasion and metastasis [207]. Although the mechanisms of MSC tropism to the tumor site are not yet fully understood, this process was exploited to develop more specific and effective anti-tumor therapies [208].

### 3.2. Angiogenesis

Numerous studies demonstrated that MSCs influenced various mechanisms of angiogenesis. Indeed, MSCs produced high levels of cytokines and growth factors, such as IL-6, IL-8, VEGF, FGF-β, PDGF, TGF-β, and angiopoietin, thus promoting tumor angiogenesis [209,210]. It was also shown that CAFs strongly mediated tumor angiogenesis by secreting immunomodulatory and pro-angiogenic cytokines and chemokines, such as IL-4, IL-6, IL-8, TNF-α, TGF-β, VEGF, and SDF-1 [69]. An in vivo study reported that the IL-6 released by MSCs led to an increase in the levels of endothelin-1 secreted in tumor cells, involving endothelial cell activation via the Akt/ERK signaling pathway promoting angiogenesis [211].

Coffman and his team also observed, in the context of an ovarian cancer model, that MSCs induced angiogenesis via a paracrine signaling loop mediated by BMP4/HH [212]. Jeon et al., in the context of ovarian cancer, reported that MSCs, in response to lysophosphatidic acid secreted by the tumor, stimulated angiogenesis via an upregulation of VEGF and SDF-1 expression [213]. In the case of “oral squamous cell carcinoma”, MSCs mediated angiogenesis by driving the excessive production of MMP-1 [214].

### 3.3. Cell Survival

Tumor sites are characterized by, among other things, hypoxia, malnutrition, and inflammation. MSCs play a crucial role in maintaining tumor cells through the release of numerous pro-survival and anti-apoptotic factors, such as VEGF, FGF-β, PDGF, TGF-β, and SDF-1α [215]. Interestingly, PDGF and TGF-β can stimulate the expression of VEGF and FGF-β, which in turn mediate the production of Bcl-2, an anti-apoptotic protein [216,217]. Also, in BM cancer cells, MSCs upregulated Bcl-2 and boosted survival rates in the context of acute myeloid leukemia [218]. In colorectal cancer, tumor cells exposed to a conditioned medium of MSCs involved a downregulation of the expression of apoptosis-related proteins Bax and p53 and an upregulation of Bcl-2. The authors also observed a significant percentage of cancer cells in the S phase [219]. Finally, Burger et al. demonstrated that SDF-1α protected leukemic cells against spontaneous apoptosis [220].

### 3.4. Metastasis

The metastasis process, which is found in advanced-stage cancer, is often associated with a poor prognosis. Schaffer et al. discriminated two distinct phases occurring during the metastasis process: The first phase involves the physical translocation of a tumor cell from the primary site through the vasculature to a distant site and the second phase concerns the ability of the tumor cell to form metastasis at this distant site [221]. Numerous studies reported the implication of MSCs in the metastatic process [218,222,223]. These effects are mediated in particular by the growth factors, cytokines, and chemokines secreted by MSCs (Figure 4). Studies evaluating the role of local resident MSCs in the tumor microenvironment reported a pro-tumorigenic role. Indeed, MSCs act like PCs and promote the extravasation of melanoma cancer cells, particularly in BM and liver [224]. Importantly, it is known that during melanoma metastasis, the skeleton and liver are part of the preferred organs for cancer dissemination with, respectively, up to 20% of patients having liver metastasis and 17% having bone metastasis. MSCs seem to regulate tumor formation and growth as well as the process of metastasizing [225,226].

As demonstrated in a mouse xenograft model of melanoma and breast cancer, MSCs secreted chemokines, namely CCL2, CCL5, CCL9, and CXCL10, which activated tumor cell migration and invasion, promoting the development of lung, bone, and lymph node metastases [158,227,228,229]. In addition, CCL5 and CCL9 chemokines led to the activation of MMPs, which induced remodeling of the ECM, promoting the migration of tumor cells from the primary site [158]. Moreover, Rodini et al. reported that paracrine signaling via TGF-β1 increased cancer cell migration and metastasis in glioblastoma [230]. Accordingly, it has also been shown in an in vitro 3D co-culture model that MSCs caused the elongation and directional migration of invasive breast cancer cells [231]. These effects were mediated through the secretion of TGF-β1 by MSCs. Additionally, Lin and co-authors demonstrated on breast cancer cells (MCF7) that MSC-derived exosomes promoted cancer cell migration through an upregulation of the Wnt and β-catenin signaling pathways [232]. Moreover, a study on hepatocellular carcinoma (HCC) models showed that HCC cells exposed to a conditioned medium of MSCs extracted from HCC were associated with an increase in cell proliferation, invasion, and metastasis through the overexpression of DNM3OS (IncRNAs) and the DNM3OS interaction with the KDM6B/TAM1 axis [233]. Importantly, MSCs also promote metastasis by activating EMT through the secretion of growth factors and cytokines, including TNF-α, HGF, EGF, PDGF, and TGF-β. These factors induced transcriptional regulators, such as Snail, Slug, Twist, and Zeb1 [234,235]. Indeed, the TNF-α and TGF-β1 secreted by MSCs increased tumor cell EMT in colon carcinoma cells via the activation of the p38 MAPK and ERK signaling pathways [236]. The effect of MSCs on the phenotype of EMT tumor cells was also observed via the differentiation of MSCs into CAFs [123,237]. The MSCs notably produced fibroblast-derived factors such as α-SMA, tenascin-C, fibroblast surface protein, IL-6, CCL5, SDF-1, MMP-2, and MMP-9 [122,159].

Finally, MSCs were able to enhance the metastasis process by physically binding to tumor cells, forming a hetero-cellular metastatic unit. Indeed, the fusion of MSCs with the cancer cells of mammary, ovarian, and lung cells were confirmed in in vitro and in vivo models [132,238]. Importantly, Fan et al. identified MSCs as being able to form a complex with tumor cells in patients with ovarian cancer [239]. Thus, MSCs, by interacting with cancer cells, give rise to cancer cells with mesenchymal phenotyping, such as CSCs and CAFs, which strongly contributes to the development of metastases through various interplays with MSCs and other components of the TME [122,132,222].

## 4. Mesenchymal Stem Cells as a Therapeutic Tool against Cancer Progression

Currently, the literature data are divided on MSCs’ therapeutic potential. As previously explained, MSCs play a dual role in cancer progression, with a pro-tumorigenic phenotype (MSCs-2) and an anti-tumorigenic phenotype (MSCs-1). In the pathophysiological context of cancer, a growing interest is occurring in the therapeutic potential of MSCs to get over the limitations of current therapy or improve their effects. MSCs have some interesting properties, such as their ability to selectively migrate to local and distant tumoral sites and penetrate the tumor stroma after intravenous injection, as demonstrated in pancreas, melanoma, lung, or breast cancer animal models [86,87,88]. As a vehicle, MSCs may deliver drugs, genes, or other therapeutic agents such as cytokines in various cancers, promoting therapeutic properties, comprising anti-tumor, -proliferative, -inflammatory, -oxidative, or -metastatic effects [240,241]. Numerous studies enumerate the potential benefic effects of MSCs transduced/transfected with viral/non-viral constructs involving the overexpression of therapeutic agents [241,242,243].

### 4.1. Resistance to Treatments

Tumor resistance to current therapies is a real problem in the fight against cancer. Indeed, the management of cancerous diseases involves several approaches, including chemotherapies, radiotherapy, targeting therapies, and, more recently, immunotherapies. These approaches can be used alone, sequentially, or concurrently. Therapies aim to promote the death of tumor cells by various mechanisms. However, tumors are able to develop ways to protect themselves from most treatments, making them resistant. This tumor resistance is due to gene alterations in tumor cells, but also the activity of the TME [244,245].

#### 4.1.1. Role of Mesenchymal Stem Cells in Tumor Resistance and Therapeutic Interest

MSCs have been shown to be partly responsible for treatment resistance through their activity in the TME [246,247,248]. It has been shown by Lis et al. that tumor-residing MSCs in ovarian cancer induced thermo-tolerance in cancer cells via activation of the SDF-1-CXCR4 axis. They also demonstrated that targeting the interaction between MSCs and cancer cells by blocking SDF-1 could restore the hyperthermia sensitivity of ovarian cancer cells [249]. Moreover, Dreuw and co-authors demonstrated that fibroblasts, resulting from the differentiation of MSCs, mediated IL-6 secretion, leading to the overexpression of Bcl-2 and Bcl-xL in tumor cells and an inhibition of apoptosis following anti-cancer treatment [250,251]. Accordingly, Gilbert et al. demonstrated that IL-6 contributed to resistance to cisplatin treatment [251]. Indeed, circulating MSCs were activated by cisplatin-based chemotherapy in tumor-bearing mouse models. The MSCs secreted two poly-unsaturated fatty acids (12-oxo-5,8,10-heptadecatrienoic acid [KHT] and hexadeca-4,7,10,13-tetraenoic acid [16:4 (n-3)]) that protect tumor cells from the cytotoxic effects of the drug [252]. Finally, Marithasan et al. examined tumors from a cohort of patients with metastatic urothelial cancer treated with an anti-PD-L1 agent (atezolizumab) and identified the main determinants of clinical outcome. The absence of response to drugs was associated with the release of TGF-β by peri-tumoral fibroblasts and an absence of CD8+ T cells in the tumor parenchyma. T cells were found in the peri-tumoral stroma, rich in fibroblasts and collagen. They also demonstrated, using a mouse model, that therapeutic co-administration of anti-TGF-β and anti-PD-L1 blocking antibodies reduced TGF-β signaling in stromal cells, allowing the release of TGF-β in the tumor center that promoted an anti-tumor immune response and tumor regression [253].

As mentioned above, MSCs have the ability to differentiate into CSCs. It is also suggested that tumor cells that undergo TEM are considered as CSCs or behave like CSCs [254,255]. These CSCs are a key factor in the resistance to therapies. Indeed, they can induce resistance to cisplatin and promote tumor relapse after the discontinuation of treatment [129]. MSCs are known to support a niche of CSCs contributing to drug resistance [256,257]. Of note is that PGE2, known to induce the autocrine expression of IL-6, IL-8, and CXCL1, promotes CSC formation [258].

Additionally, Skolekova and co-authors demonstrated that the pre-exposure of MSCs to cisplatin altered the phosphorylation of several tyrosine kinases, including STAT3, c-Jun, WNK-1, and p53, promoting the production of IL-6 and IL-8 and MSC survival, leading to tumor cell chemoresistance [259]. On the other hand, Kabashima et al. reported that CAFs maintained CSCs by secreting the Notch ligand Jagged-1, thereby promoting drug resistance [260].

#### 4.1.2. Radioresistance and Mesenchymal Stem Cells

Radiotherapy is a treatment modality that can be used to treat about 60% of patients with cancerous diseases. Radiation therapy, known as a great technological progress over the past 20 years, allows a more precise delivery of radiation while sparing healthy tissue surrounding the tumor. Nevertheless, the radioresistance of certain cancers remains a major problem with this therapeutic application. A main factor leading to radioresistance is linked to CSCs’ activities in the TME. CSCs are dramatically implicated in the metastasis process, relapses, and the failure of radiotherapy, and are associated with a poor prognosis for patients [261,262]. The intrinsic radioresistance of CSCs can be attributed to several factors [263]. In the setting of breast cancer, Philipps and his team associated the radioresistance of CSCs with a lack of oxidative stress generated due to the high ability of CSCs to scavenge free radicals and activate mechanisms of DNA repair [264]. Also, other studies demonstrated a repopulation of CSCs after radiotherapy through the activation of the Wnt/β-catenin signaling axis that promotes self-renewal [265]. Moreover, in the context of mucoepidermoid carcinoma, CSCs’ radioresistance was associated with the activation of the NFκB signaling pathway [266]. In addition to these mechanisms, several studies reported the activation of survival signaling pathways, such as the anti-apoptotic Bcl-2 protein and PI3K/Akt/mTOR signaling pathways, which also contributed to CSCs’ radioresistance [266,267]. Radiation therapy can also induce EMT in tumor cells, thereby increasing their motility and invasive abilities in several cancers, including breast, lung, liver, and glioma [268,269,270].

### 4.2. Mesenchymal Stem Cells as a Therapeutic Vector

**Figure 6:**
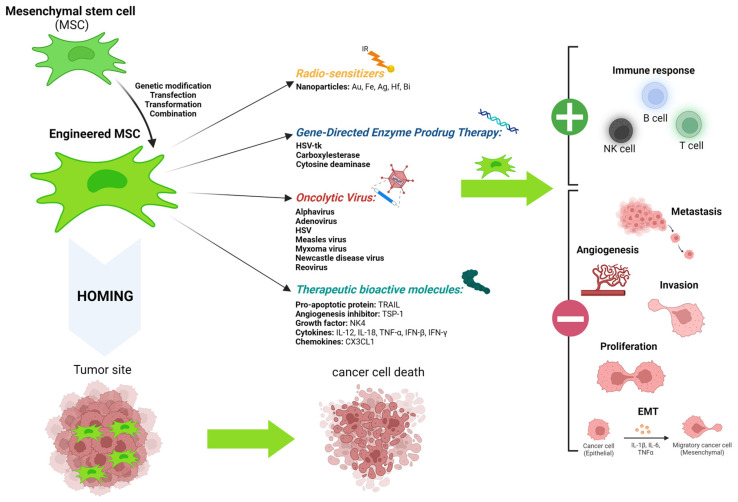
Mesenchymal stem cells as vectors for therapeutic agents. Ag: silver; Au: gold; CX3CL1: C-X3-C motif chemokine ligand 1; EMT: epithelial–mesenchymal transition; Fe: iron; Hf: hafnium; HSV-tk: herpes simplex virus thymidine kinase; IFN: interferon; IL: interleukin; MSC: mesenchymal stem cell; NK cell: natural killer cell; TNF-α: tumor necrosis factor-α; TRAIL: TNF-related apoptosis-inducing ligand; TSP-1: thrombospondin-1. (Figure designed by BioRender).

Over the past two decades, interest in MSCs was focused on their potential to migrate to tumor sites. Nevertheless, research teams remain cautious about their use due to their predominantly pro-tumorigenic characteristics. However, the inherent tumor tropism of MSCs gives them an interesting selective power for depositing anti-tumor agents thereon. Also, the development of genetic engineering techniques has allowed the control of the release of anti-tumor agents in situ [271,272,273]. These agents are, in particular, proteins, suicide genes, nanoparticles, or even oncolytic viruses, as presented in Figure 6. Of note is that an increasing number of studies reported that MSC-derived exosomes can also be used as anti-tumor agents. The approach around exosomes will not be developed in this present review.

#### 4.2.1. Types of Vectoring Making Use of Mesenchymal Stem Cells

1. Therapeutic macromolecules

The therapy with bioactive molecules was used for the first time in 2002, with a modification involving an overexpression of IFN-β by engineered MSCs [274]. Afterwards, MSCs were modified to express cytokines, chemokines, interferons, and pro-apoptotic or anti-angiogenic factors, as presented on Figure 6. MSCs are able to deliver a variety of cytokines and growth factors to the tumor site that were identified as anti-tumor agents, comprising IFN-α, IFN-β, IFN-γ, IL-2, IL-7, IL-12, IL-15, IL-18, IL-25, and NK4, an antagonist of HGF [208,275,276]. Mirlekar and co-authors, in an in vivo study on renal cell carcinoma and cervical tumors, showed that MSCs expressed IL-12 and inhibited tumor proliferation [277,278]. Moreover, IL-12 is able to activate T cells and cytotoxic NK cells [279]. A particular interest was also taken in the anti-tumorigenic effect of the factor-related apoptosis-induced ligand (TRAIL) [99,280,281].

2. Gene-directed enzyme prodrug therapy: suicide genes

A therapeutic approach using MSCs is based on genetic modifications allowing them to express suicide genes [282]. Among the strategies used for an anti-tumor role based on suicide genes and delivered by MSCs, we can cite three majors factors used: TK (GCV), cDy::UPRT (5-FC), and CD (5-FC) [243,275,282,283].

An in vivo study on prostate cancer used MSCs designed to express cytosine deaminase::uracil phosphoribosyltransferase (CD::UPRT), which is a transgene produced from suicide gene expression. They showed that modified MSCs inhibited tumor growth. Indeed, CD::UPRT was able to convert the relatively non-toxic 5-fluorocytosine (5-FC) into toxic 5-fluorouracil (5-FU) [284]. Another in vivo study on colon cancer demonstrated the same effectiveness concerning genetically modified MSCs [285]. Moreover, in the treatment of bone metastases, Segaliny and co-authors demonstrated that MSCs designed to overexpress P-selectin glycoprotein ligand-1 (PSGL-1)/Sialyl-Lewis X (SLEX) and modified versions of cytosine deaminase (CD) and osteoprotegerin (OPG), had better homing toward metastatic bone tumor cells, possibly due to interactions between the PSGL-1/SLEX expressed by MSCs and the P-selectin expressed by endothelial cells at the vascular level of the tumor. Thus, the authors showed that modified MSCs could effectively eliminate tumor cells while preserving bone integrity [286].

3. Oncolytic virus

Another strategy in the development of anti-tumor MSCs consists of using cells to deliver an oncolytic virus (OV) [287]. Indeed, over the past 20 years, after that oncorine was validated as the first virus drug [288], OVs were studied as therapy in the fight against cancer [289,290,291]. This new anti-tumor approach is based on the use of viruses able to replicate inside tumor cells to kill them. Nevertheless, not all viruses can be used as OV; they need to have particular characteristics, such as the ability to specifically target tumor cells, low toxicity, a significant capacity to destroy tumor cells by viral proliferation, an oncolytic and targeting potential enhanced by structural and genetic modification, as well as the ability to carry transgenes in order to elevate their therapeutic potential [292,293]. Thus, several viruses from various viral families were studied [289,294]. As reported by Darestani et al., numerous preclinical studies demonstrated efficient features for MSCs as OV carriers. Among the OVs that can be transported by MSCs, we could cite adenovirus (Ad), herpes simplex virus (HSV), measles virus (MV), myxoma virus (MYXY), Newcastle disease virus (NDV), reovirus [295,296], as well as alphaviruses [24,297].

This therapeutic approach mainly exerts its effect through the direct oncolysis caused by the virus cell proliferation and tissue spreading [298]. Subsequently, the OV will promote antiviral immune responses (e.g., Interferon type I), leading to the death of infected tumor cells. Nevertheless, OV treatment largely depends on their ability to reach the tumor site without being recognized by the immune system [299,300,301,302,303,304].

In a study on glioma, the authors reported that NDV-carrying MSCs enhanced apoptosis and inhibited tumor cell proliferation and that the TRAIL protein played an important role in the cytotoxicity of the MSCs carrying NDV [305]. Moreover, a study on ovarian cancer demonstrated that groups treated with MSCs carrying MV had better survival rates compared to groups treated with the virus alone [306]. Interestingly, Zhan et al. also showed in glioma that MSCs carrying oncolytic recombinant adenovirus enriched with IL-24 and endostatin, and regulated by doxycycline, increased the anti-tumor response by inhibiting tumor growth [307]. It has also been demonstrated in glioma that MSCs carrying CRAD (CRAD-S-pk7), which is an adenovirus, potentialized anti-tumor activities and induced a significant decrease in tumor volume compared to the group administered with the virus alone [308,309]. M1 is an oncolytic alphavirus which has been validated for possible treatments of breast and brain cancers [310].

4. Metallic nanoparticles

Recently, radiosensitizing agents coupled to MSCs demonstrated interesting anti-tumor properties [311]. Indeed, in order to sensitize tumor cells to ionizing radiation and to counteract cell radioresistance, metallic nanoparticles (NPs) exhibiting elements with a high atomic number (Z), such as gold (Au), hafnium (Hf), or bismuth (Bi), were used as radiosensitizers [312,313,314,315]. Nanoparticles could be used to potentialize radiation toxic effects. Indeed, they can accumulate in tumor areas by passive targeting through the enhanced permeability and retention (EPR) effect, by active targeting mediated by ligand–receptor interaction, or by a combination of passive and active targeting [316,317]. However, the therapeutic results of these new nano-radiosensitizers remain problematic in the case of inadequate targeting [318,319]. The non-viral strategy using nanoparticle-based gene delivery via bioengineer stem cells could offer a new theragnostic perspective. Interestingly, Huang and team used a magnetic ternary nanohybrid (MTN) system carried by superparamagnetic iron oxide nanoparticles to construct MSCs expressing TRAIL using magnetic force and receptor dual targeting. They imaged the MTN-transfected hMSCs using MRI and observed an apoptosis activation as well as a decrease in the cancer progression and an increase in the survival rate on the mice model of glioma [320].

#### 4.2.2. Therapeutic Potential of Mesenchymal Stem Cells in the Case of Melanoma

Melanoma is a malignant tumor of melanocytes (derived from the neural crest) that represents the third most common cutaneous malignancy. Globocan estimated 325,000 new cases in 2020, 1.7% of global cancer diagnoses [321]. Melanoma management at an early stage leads to 93.5% 5-year relative survival, with the most common management coming via surgical resection. However, depending on the cancer stage at diagnosis, the prognosis can become poorer due to a high risk of melanoma metastasis beyond its primary site. Indeed, metastatic melanoma is the most mortal type of cancer with a 5-year relative survival reaching 4.7% in the worst cases, when the diagnosis is completed on older age patients for a distant melanoma [322]. This low survival rate is mainly due to anticancer agent resistance [323]. Indeed, it is important to consider the immunologic and genetic profile of patients to choose the most appropriate therapy or combination of therapies in order to keep the clinical response as long as possible [324,325].

Numerous studies reported the promising effects of genetically modified MSCs in order to overexpress therapeutic agents. A study reported that MSCs designed to express TNF-α could trigger the caspase-3/-7-dependent apoptosis of human A375 melanoma cells. In this study, the authors showed that a subcutaneous injection of A375 melanoma cells and genetically modified MSCs, in a ratio of 1:4, could induce 97.5% tumor regression in mice [326]. Interestingly, Petrov et al. used adenovirus comprising six vectors encoding IFN-α in a lung metastasis model of melanoma and demonstrated that modified MSCs significantly reduced the growth of melanoma cells and significantly prolonged the survival of mice through an increase in apoptosis and a decrease in proliferation and angiogenesis [327]. It has also been demonstrated that MSCs expressing thrombospondin-1 (TSP-1) in a metastasis melanoma model decrease nodule apparition and exerted an anti-tumor effect via an activation of T cells, an upregulation of Bax pro-apoptotic protein, and a downregulation of Bcl-2 anti-apoptotic protein [88]. Importantly, the interest in using MSCs as a vehicle is their capacities to target tumor stroma combined with their longevity in the tumor microenvironment as well as their lineage differentiation [52].

Another study reported the interesting role of MSCs derived from adipose tissue as a tool for delivering therapeutic genes to tumor cells. MSCs were used to deliver an anti-cancer drug, namely cisplatin, coupled with a lentiviral vector expressing IFN-β cytokine for its anti-proliferative, immunomodulatory, and anti-angiogenic activities [328]. MSCs genetically designed to produce IFN-β have been used against other cancers and demonstrated interesting effects. Authors reported anti-proliferative and pro-apoptotic effects on tumor cells [329]. It has been shown that IFN-β was able to inhibit the proliferation of melanoma tumor cells and to modulate the immune response [330]. In addition, a tumor-specific antibody coupling with IFN-β has been shown to be more effective than antibody treatment alone [331]. Finally, Yang et al. demonstrated that sequential IFN-β and cisplatin treatment improved the adaptive immune response [332].

Authors demonstrated in a mouse melanoma model that the association of therapeutic agents led to an increase in survival rate and inhibited the growth of melanoma compared to the control group and the group treated with cisplatin alone. Accordingly, other articles report the synergic effect between therapeutic agents and MSCs’ protective properties [283]. However, studies using MSCs alone exposed their beneficial effects, such as low immunogenicity and immunosuppressive, anti-tumor, anti-proliferative, anti-inflammatory, or antioxidant properties, depending on the model studied [7,333]. MSCs can induce a phenotype reprogramming of microenvironment resident cells, such as macrophages, in a mouse model of melanoma injected with xenogeneic bone marrow MSCs [334].

Çakır and co-authors reported similarities in the phenotype and function of MSCs and melanoma-associated fibroblasts (MAFs) [335]. Indeed, they observed an increase in macrophage-secreted IL-10 under the control of MAFs which were in intimate contact with macrophages in excised melanoma from two different patients. The MAFs caused immunosuppression by promoting the phenotype reprogramming of macrophages. The authors also showed that COX and IDO were implicated in this immunomodulation and demonstrated their critical role in cancer-progression-promoting immunosuppression. The inhibition of COX-1, 2, or IDO led to limited MAF–macrophage interactions and the modulation of intra-tumoral immune responses. The use of MSCs as vectors for COX and/or IDO inhibitors could be strategic for treating melanoma patients.

Importantly, MSCs as a deliverer of gene-directed enzyme prodrug molecular chemotherapy could also open a new era in therapeutic approaches using suicide genes, by avoiding the gastrointestinal toxicities related to chemotherapeutic drug absorption, such as the 5-Fluorouracil. Kucerova and co-authors explored the effect of a combination of cytosine deaminase with prodrug 5-fluorocytosine, delivered by MSCs derived from adipose tissue [336]. In their study, they selectively produced a cytotoxic agent in situ that caused a complete tumor regression in 89% of tumors and a tumor growth inhibition on a melanoma mice model co-injected with modified MSCs and tumor cells. In an in vivo model of lung metastases of melanoma, the MSCs carrying a recombinant oncolytic MYXY virus expressing IL-5 improved animal survival rate. Interestingly, the authors reported, concerning the anti-cancer mechanism of the proposed therapy, an increase in the level of natural killer (NK) cells recruited during the activation of CD8+ T cells. Moreover, mice who received MSCs coupled to the OV exhibited an increase in the release of pro-inflammatory cytokines, namely IL-15, IL-1β, IFN-γ, TNF-α, PD-1, and PD-L1, compared to mice who received virus alone [285].

Moreover, the therapeutic effects of BM-derived MSCs expressing CMV-thymidine kinase (TK) on pulmonary melanoma metastasis combined with the prodrug ganciclovir were investigated [243]. The MSCs were engineered through a non-viral gene vector. In vitro results displayed that the engineered MSCs caused a significant anti-tumor effect in the presence of ganciclovir in a dose-dependent manner. In vivo studies confirmed these beneficial effects on a metastasis tumor model.

However, the literature data report contradictory results about role of MSCs in tumor progression. The ambivalent role of MSCs in tumor suppression and progression needs to be understood before envisaging their use as cancer therapy. Indeed, depending on the cancer types or research models used, MSCs exhibit pro- and anti-tumorigenic potential depending on their interplays with resident cancer cells in the microenvironment. Importantly, MSCs injected as a vehicle for therapeutic agents or injected alone exhibit a protective role and anti-tumor effect in most in vivo and in vitro studies about melanoma cancer. These therapeutic strategies could be really promising for targeting tumor cells and help in the case of radioresistance for all types of resident cells in tumor parenchyma, particularly concerning CSCs.

#### 4.2.3. Therapeutic Potential of Mesenchymal Stem Cells in the Case of Lung Cancer

The Globocan epidemiologic study reported 2,206,771 new cases of lung cancer in 2020, i.e., 11.4% of all identified cancer cases. Lung cancer is the most common cancer and the deadliest worldwide, with the number of deaths from lung cancer reaching 18% in 2020 [321]. There are different types of lung cancers, namely non-small-cell lung cancer (about 80% to 85% of all lung cancers), small-cell lung cancer (about 10% to 15% of cases), and other tumors occurring in the lungs (5% of cases). Currently, lung cancer management includes surgery, radiotherapy, chemotherapy, targeted therapy, or immunotherapy. Despite the combination of major therapeutic approaches, the 5-year survival of non-small-cell lung cancer remains at 8–15% [337]. The dramatic incidence of lung cancer, as well as the poor prognosis, pushes health professionals and researchers to imagine new therapeutic approaches to improve lung cancer management.

Various studies demonstrated that MSCs promoted tumor growth, metastasis, and neovascularization during lung cancer [156,338,339]. Thus, MSCs used as vehicles for therapeutic agent delivery represent a seductive strategy and a tool to target tumor sites, considering their homing capacity [340]. Importantly, engineered MSCs labeled with superparamagnetic iron oxide nanoparticles were used to track MSC localization via MRI [341]. It has been reported that after the intravenous injection of superparamagnetic-iron oxide-loaded MSCs in a multiple lung metastasis mouse model, modified MSCs were localized in tumor sites. The homing ability of MSCs makes them good candidates as vectors for therapeutic agents. It has been demonstrated in numerous studies that the expression of TRAIL by MSCs induced apoptosis of tumor cells in the context of colorectal, lung, breast, brain, and cervix cancers [342,343,344]. Interestingly, the most studies on the anti-tumor role of engineered MSCs in lung cancer relate to TRAIL. Loebinger et al. hypothesized that MSCs modified to produce and deliver TRAIL at tumor sites, a transmembrane protein that mediates apoptosis, could cause the death of tumor cells [343]. The authors engineered human MSCs transduced with TRAIL and the IRES-eGFP reporter gene, under the control of a tetracycline promoter using a lentiviral vector, and tested their anti-tumor effect on a lung cancer model. The MSC model homed to tumor sites, significantly reduced tumor growth, and completely cleared the metastatic disease in 38% of mice with tumors compared to 0% of controls.

Moreover, another study targeted MSCs to abrogate their deleterious initial role in cancer progression by silencing their TGF-β1 expression, which reverted their exosome-mediated EMT and enhanced the anti-proliferative and pro-apoptotic effect of MSCs in lung cancer [345]. By blocking TGF- β1 expression, they deactivated the Smad2/3, Akt/GSK-3β/β-catenin, NF-κB, ERK, JNK, and p38 MAPK signaling pathways, which are known to promote cancer progression. Moreover, mouse MSCs transduced with adenoviral vector expressing CX3CL1, injected into mice bearing lung metastases, mediated a decrease in the development of lung metastases and an improved survival rate [346]. The anti-tumor effects reported were related to both innate and adaptive immune modulation through an increase in infiltration of CD8+ lymphocytes, CD4 lymphocytes, and NK cells.

It has also been demonstrated that MSCs loaded with a doxorubicin (DOX)–polymer conjugate were able to penetrate tumor sites, inhibit tumor growth, and exhibit better efficacy than the drug alone [347]. Major studies on the anti-tumor role of engineered MSCs are related to pro-apoptotic protein delivery. Finally, Hakkarainen et al. explained that systemic adenoviral delivery lacked efficacy due to liver sequestration of the intravenously administered virus [348]. They used MSCs transduced by the OV to avoid liver sequestration. They noted in an in vivo model of lung cancer that intravenously injected-MSCs homed primarily to the lungs, that the virus delivered by the MSCs was released into advanced lung tumors enhancing mice survival rate, and that the same dose of the virus injected alone was found in the liver. MSCs are of high interest for their homing capacity as well as for improving the bioavailability of systemically administered oncolytic adenoviruses. Importantly, Ando and co-authors designed a suicide system via MSCs based on an inducible caspase-9, activated using a specific chemical inducer of dimerization (CID), for adenoviral-based delivery to lung tumors to sensitize non-small-cell lung cancer to apoptosis [349]. Their engineered MSCs coupled with CID-activated iC9 were combined or not with bortezomib, an inhibitor of the proteasome. The combination of the MSC-based delivery of the iC9 suicide gene to human non-small-cell lung cancer and bortezomib enhances the anti-tumor activity and the addition of bortezomib offered a greater reduction of tumor size.

## 5. Conclusions

MSCs play a major role in cancerous diseases. Understanding their behavior in this setting may be the key to the fight against cancer. Nevertheless, their use has long been debated due to all the diverging results about MSCs and cancer. The literature data describe both anti- and pro-tumorigenic effects. Moreover, the tumor microenvironment is a heterogeneous complex, and a dynamic system comprising stromal cells with different morphological and functional activities. Despite this reality, the latest therapies based on MSCs bring new hope to cancer patients by offering more effective anti-tumor treatments. Indeed, the use of MSCs as vectors for therapeutics represents an important advance for more specific and personalized treatments. Currently, it seems crucial to better understand the interactions between the different subsets of MSCs, cancer cells, as well as tumor microenvironment components to propose highly specific therapeutic approaches. From a pharmacological point of view, it may be important to address the drug treatment that may favor the phenotype switch between the different subsets of MSCs (e.g., MSCs-1 versus MSCs-2) and/or dedifferentiation into physiological PC-like cells.

## Figures and Tables

**Table 1:** Canonical surface markers used to identify mesenchymal stem/stromal cells in vitro.

Expressed Surface Markers	Negative Selection Surface Markers	Characteristics
CD105	CD11b	Plastic adherence
CD90/THY-1	CD14	Multi-lineage differentiation
CD73	CD19	Plasticity
CD44	CD34	
CD166	CD45	
CD29	CD79a	
STRO-1	HLA-DR	
CD146/MUCIN		
CD248/ENDOSIALIN		
CD140β/PDGFRβ		

CD: cluster of differentiation; HLA-DR: human leukocyte antigen-DR isotype; PDGFRβ: platelet-derived growth factor receptor β; STRO-1: stromal cell surface marker-1.

## Abbreviations

Ad: adenovirus; AKT: protein kinase B; ANPEP: alanyl aminopeptidase; α-SMA: α-smooth muscle actin; Bcl: B-cell lymphoma/leukemia; BM: bone marrow; BMP4/HH: bone morphogenetic protein 4/hedgehog; C3a: cleaved complement component 3 anaphylatoxin; CAAs: cancer-associated adipocytes; CAFs: cancer-associated fibroblasts; c-AMP: cyclic adenosine monophosphate; CCL: CC chemokine ligand; CD: cluster of differentiation; CNS: central nervous system; COL: collagen; Cox-2: cyclooxygenase-2; CSCs: cancer stem cells; CSF1: macrophage colony-stimulating factor 1; Cspg4: chondroitin sulfate proteoglycan 4; CXCL: C-X-C motif ligand; CXCR: C-X-C motif chemokine receptor; DDR2: discoidin domain receptor tyrosine kinase 2; DUSP1: dual-specificity phosphatase 1; ECM: extracellular matrix; EGF: epidermal growth factor; EMT: epithelial–mesenchymal transition; EndEMT: endothelial–mesenchymal transition; EPR: enhanced permeability and retention; ERK; extracellular signal-regulated kinase; FASL: fas ligand; FGF-β: basic fibroblast growth factor; FSP1: fibroblast-specific protein 1; GDNF: glial-cell-line-derived neurotrophic factor; GF: growth factor; GM–CSF: granulocyte–macrophage colony-stimulating factor; HCC: hepatocellular carcinoma; HGF: hepatocyte growth factor; HIF-1: hypoxia-inducible factor-1; HLA-DR: human leukocyte antigen-DR isotype; HSV: herpes simplex virus; IDO: indoleamine-2,3-dioxygenase; IFN: interferon; IGF: insulin growth factor; IL: interleukin; iNOS: inducible nitric oxide synthase; ISCT: international society for cellular therapy; JAK: janus kinase; lncRNAs: long non-coding RNAs; MAFs: melanoma-associated fibroblasts; mIR/miRNA: microRNA; MMPs: matrix metalloproteinases; MSC(s): mesenchymal stem cell(s); MTN: magnetic ternary nanohybrid; mTOR: mammalian target of rapamycin; MV: measles virus; MYXY: myxoma virus; Mφ: macrophage; NDV: Newcastle disease virus; NFkB: nuclear factor-κB; NG2: neural/glial antigen 2; NK: natural killer; NO: nitric oxide; NPs: metallic nanoparticles; OV(s): oncolytic virus; p38 MAPK: p38 mitogen-activated protein kinase; PCs: pericytes; PD-1: programmed cell death-1; PDGF: platelet-derived growth factor; PDGFRβ: platelet-derived growth factor receptor β; PD-L1: programmed cell death ligand 1; PGE2: prostaglandin E2; PI3K: phosphoinositide 3-kinase; pMSCs: perivascular mesenchymal stem cells; PSGL-1: p-selectin glycoprotein ligand-1; RANTES: regulated on activation, normal T cell expressed and secreted; SCF: stem cell factor; SDF-1: stromal cell-derived factor 1; Smad: small mothers against decapentaplegic; Src: proto-oncogene tyrosine-protein kinase; STAT: signal transducers and activators of transcription; STRO-1: stromal cell surface marker-1; TAFs: tumor-associated fibroblasts; TAM1/2: tumor-associated macrophage 1/2; TAN1/2: tumor-associated neutrophil 1/2; TEM1: tumor endothelium marker 1; TGF-β: transforming growth factor-β; TGFBR: transforming growth factor b receptor; TME: tumor microenvironment; TNF-α: tumor necrosis factor-α; TNFR: tumor necrosis factor receptor; TRAIL: tumor-necrosis-factor-related apoptosis-inducing ligand; VCAM: vascular cell adhesion molecule; VEGF: vascular endothelial growth factor; vFB: vascular fibroblasts; vSMCs: vascular smooth muscle cells.
